# Oxygenator failure – Are you prepared?

**DOI:** 10.1051/ject/2023020

**Published:** 2023-09-08

**Authors:** Tim Willcox

**Affiliations:** 1 Editor, ANZCP PIRS-2; 2 Auckland City Hospital 2 Park Road Grafton 1023 Auckland New Zealand; 3 Department of Anaesthesiology, Faculty of Medical and Health Sciences, University of Auckland Auckland New Zealand

In the last six months, the frequency of Australia and New Zealand Perfusion Incident Reporting System (ANZCP PIRS) reports has been about one per fortnight which is representative of the rate of reports over the last 3 years. The mix of severity is 50:50 Good Catch Near-Miss and Good Catch No-Harm (reached the patient with no discernible harm occurring). While we continue to encourage voluntary reporting to PIRS of Good Catch near-miss and no-harm incidents, arguably changing the blame culture associated with unintended events to that of the benefits of sharing the lessons from the smart workarounds of reporting Good Catch incidents lies with perfusion leadership. How frequently do you hear the comment – “oh that happened to…me us them”.

In the last six months somewhat interestingly there have been two reports of oxygenator change-out during CPB (one in process at the time of writing) as well as a decision not to change out but manage a leaking oxygenator. Oxygenator change-out is considered a rare event and many perfusionists will say they have never changed out an oxygenator in their career. A more interesting question would be how often was change-out considered but on a risk-benefit basis the decision was made to continue with the “faulty device”? There are a number of PIRS reports in the last 2 years where the oxygenator was changed-out either immediately prior to CPB or in one case where the patient was weaned from CPB prior to cross-clamping to enable changeout. An interrogation of the FDA Manufacturer and User Facility Device Experience (MAUDE) database (https://www.accessdata.fda.gov/scripts/cdrh/cfdocs/cfmaude/search.cfm) reveals multiple instances of oxygenator change-out in the last 12 months. Furthermore, there were many instances where change-out was considered but not done, sometimes with periods of marked hypoxaemia (“They continued to use the involved product and finished the case with it. However, they were unable to get the desired po2 while using it during the case”).

So, what determines the threshold for changing out a “failing” oxygenator? Clearly, the elimination of causes apart from the device itself with a clear analysis pathway for the diagnosis of oxygenator failure is required to preclude unnecessary changeout. For instance, gas supply issues related to a mis-seated vaporiser have been commonly reported to PIRS. Alan Soo and colleagues, in their 2012 paper Successful Management of Membrane Oxygenator Failure during Cardiopulmonary Bypass -The Importance of Safety Algorithm and Simulation Drills [1] make the following comment in the discussion:

“Groom et al. proposed replacement of the failed oxygenator by inserting a second oxygenator in parallel within the cardiopulmonary bypass circuit obviating the need to stop CPB (5). However, as we are not familiar with this technique, it was not used in this case. In our center, the perfusion staff perform simulation drills on a weekly basis for management of emergency situations, which includes oxygenator replacement. We feel that this practice has enabled the safe and smooth replacement of the oxygenator in this case. This is supported by Darling and Searles, who suggested that written protocols with simulation practice are important in improving efficiency in emergency situations (6). Therefore, we propose that all cardiac surgery departments should be aware of these incidents and an algorithm should be put in place and adhered to with regular simulation drills to improve efficiency if such a situation were to occur.”

The two points of interest from this comment are (a) that the authors were not familiar with the technique described by Groom et al. in 2002 [2] for parallel replacement of the oxygenator (the PRONTO technique), and (b) that at their centre the perfusion staff perform simulation drills on a weekly basis for management of emergency situations. It is plausible that there are few centres in Australasia (or indeed anywhere) that perform simulation drills every week. Therefore, given the infrequency of oxygenator failure, the threshold for changing an oxygenator is likely to be very high, especially in environments with no perfusionist assistance on site. That begs the question why is the PRONTO line or similar arrangement not universal? As Gary Grist states in an article in Perfusion Theory, “By this time, all perfusion programs should be using a PRONTO line which enables an oxygenator to be changed out without taking the patient off CPB” [3]. PIRS has previously included a 2011 YouTube video by Bob Groom of the PRONTO changeout process with a report of oxygenator changeout and it is easily modifiable for differing CPB configurations (https://www.youtube.com/watch?v=kehXUDGjZj8). This straightforward technique avoids any time constraint of separation from CPB during the procedure. Exposure to a bad outcome associated with interruption of CPB to change an oxygenator could be difficult to justify to a subsequent enquiry where this safer alternative was available.
